# The Specialist

**Published:** 1920-06-19

**Authors:** 


### THE SPECIALIST.

ALMOST daily we read of the danger to medical research, and indeed to every other branch of scientific work, which lurks in the tendency among often brilliant men to think only in their own particular sphere of expert knowledge. Such isolation where it exists, the resulting lack of real co-operation, and all that this implies are, of course, utterly to be condemned, but are we quite justified in saying that the day of the "specialist surgeon" and the "specialist physician" is past? Such words written where they meet the public eye are not a little likely to be completely misunderstood by the layman. Among all things medical and most professions, we find the bad mixed with the good. And in the medical world, if a man practises and thinks in one particular line alone, providing the spectacle of a scientist who intentionally rings himself round with a wall preventing not only his perception of the work of others, but also their perception of his, then he is of but small value to the ideals of research.

But we doubt whether it is justifiable to lead the public, particularly those who give to charities, to assume that this description of circumscribed activity can be applied to our many practising "specialists," as, for want of a better term perhaps, the average patient dubs the great army of men who form the honorary staffs of our greater hospitals. In a great deal of the recent publicity given to these matters there is a strong suggestion running through the wording of press criticisms, that each of these men is concentrated, almost knowingly so, in his own particular sphere.

Now we know, and the public should know also, that this situation has rarely existed, and if it ever has, the fault has not been with the men themselves. We deny absolutely that the "physiologist," for example, has, either of his own accord or through the obtuseness of the "physician," been shut away from participation in the general work of caring for mankind. The first point to be recalled is that the professional men under discussion have all had a long, difficult and exacting training, the first nine-tenths of which has been practically identical for each. Later they have developed special facility and gained special experience in certain types of disease and methods of constructive thought. They are all practically and highly experienced and qualified in all branches of medical science. The very process of their election to the hospital staffs *via* the lanes of the keenest competition automatically provides that this is so, and it would, we think, be hard to find a modern first-class physician who has not the latest teachings of physiology well to the forefront of his mind. To the laymen the very practice of etiquette by the specialist, when he refuses an opinion upon a condition in disease which does not come within the scope of his avowed practice, tends to disseminate the idea of the water-tight compartment. But hospital practice, research practice, is a very different matter to that of the private consulting-room, and it has been our own experience in attending the periodic meetings which research societies frequently hold, that the discussion is readily followed into any medical channel by the majority of the speakers, whatever be their avowed "speciality." The public must not too readily agree that the lack—or, more accurately, slowness—of research co-ordination in the past has been due to the faulty attitude of the individual scientist or doctor. The vital factor has been the lack of *available means* for such co-ordination. This deficiency has been noticeable everywhere, whether we consider the matter as an international or national problem. The gulf has been formed by a type of administration which, whatever else were its ultimate aims, did not place investigational matters on a "team work" principle. The personal material, the knowledge and the experience has been there year by year, but largely unguided, and until the great awakening of 1914, many sections had tended to drift further and further apart. More recently, on every hand, in nations, in hospital, in universities, even in our small provincial towns, we see a new administration arising, a phase of concerted effort. This week we have to record the munificence and value of the Rockefeller gift.

